# The influence of Si(iv) on the reactivity of [≡Fe(iii)]/[≡Fe(ii)] couples for 2-nitrophenol reduction in γ-Al_2_O_3_ suspensions†

**DOI:** 10.1039/c7ra13201h

**Published:** 2018-02-15

**Authors:** Liang Tao, Shan-Li Wang, Fang-Bai Li, Nin-Ya Yu, Ke Wu

**Affiliations:** Guangdong Key Laboratory of Integrated Agro-environmental Pollution Control and Management, Guangdong Institute of Eco-environmental Science & Technology Guangzhou 510650 P. R. China cefbli@soil.gd.cn +86-20-87024123 +86-20-37021396; Department of Agricultural Chemistry, National Taiwan University Taipei 10617 Taiwan Republic of China; National & Local Joint Engineering Laboratory for New Petrochemical Materials and Fine Utilization of Resources, Hunan Normal University Changsha 410081 P. R. China

## Abstract

In a natural environment, Fe(ii) adsorbed onto the surfaces of natural particles to form various surface complex species can influence the transformation of contaminants. The reductive reactivity of the [≡Fe(iii)]/[≡Fe(ii)] couples are close correlated with the surrounding conditions. In this study, we investigated the effects of Si(iv) on the reductive reactivity of [≡Fe(iii)]/[≡Fe(ii)] couples adsorbed onto γ-Al_2_O_3_. Experiments were conducted under different conditions to investigate the effects of Si(iv) on the reactivity of [≡Fe(iii)]/[≡Fe(ii)] couples for 2-nitrophenol (2-NP, selected as the model pollutant) reduction in γ-Al_2_O_3_ suspensions. Kinetics results revealed that chemical adsorption is the rate limiting step in Fe(ii) and Si(iv) adsorption processes and the reduction of 2-NP is an endothermic reaction. The linear correlations between the reduced peak oxidation potential (*E*_p_) (*versus* SCE) and 2-NP reduction rate (ln *k*), and between the adsorbed Fe(ii) density (*ρ*_Fe(II)_) and ln *k*, illustrated that *E*_p_ and *ρ*_Fe(II)_ are two key factors in the inhibiting effects of Si(iv) on the reductive reactivity of Fe(iii)/Fe(ii) couples on γ-Al_2_O_3_. The results of Fe *K*-edge X-ray absorption spectroscopy revealed that the increase of Si(iv) concentration resulted in the gradual change in the composition of the adsorbed Fe species from pure ≡AlOFe^+^ (γ-Al_2_O_3_ surface-bound Fe(ii) species with higher reductive reactivity) to a mixture of ≡AlOFe^+^ and ≡SiOFe^+^ (SiO_2_ surface-bound Fe(ii) species with lower reductive reactivity), leading to the decrease in *ρ*_Fe(II)_, the positive shift in *E*_p_, the increase in activation energy (*E*_a_), and consequently the decrease in the reduction rate (ln *k*) of 2-NP.

## Abstract 2

In a natural environment, Fe(ii) adsorbed onto the surfaces of natural particles to form various surface complex species can influence the transformation of contaminants.

## Graphical abstract

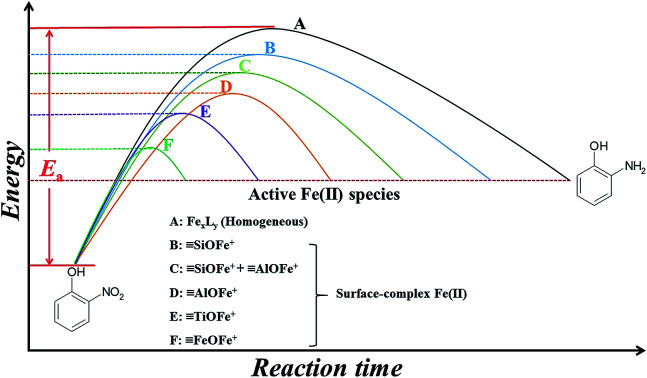

## 1. Introduction

Nitroaromatic compounds and their derivatives (NACs) are highly toxic and recalcitrant chemicals, and have been listed as priority pollutants.^1^ They are widely used in the production of chemical products, such as chemical intermediates, pesticides, herbicides, synthetic dyes, and explosives,^2^ leading to their widespread occurrence in the environment. The environmental fates of NACs are determined by many factors,^1,3–6^ among which, mineral-bound [≡Fe(iii)]/[≡Fe(ii)] couples have been proven to play a critical role in the reduction of NACs into the corresponding nitroso/amino compounds under abiotic conditions.^7,8^ The general consensus is that aqueous Fe(ii) can be adsorbed onto mineral surfaces and stabilized by surface hydroxyl groups. The resultant Fe(ii) surface complexes have lower redox potentials compared to their aqueous counterparts.^9–12^ Previous studies have found that the reductive reactivity of the Fe(ii) surface complex are dependent on reaction pH,^8,13,14^ reaction temperature,^7^ co-existence organic compounds,^5,6,15,16^ the properties of adsorbing surfaces,^4,17^ soil use types,^18^ and so on.

Silica, aluminum and iron are the second, third and fourth most abundant elements in the Earth's crust, respectively. The fraction of alumina in clays is relatively high varying from very low to 75%. γ-Al_2_O_3_ is an important class of Fe-free minerals, with the ability of allowing the adsorption of Fe(ii) over a wide range of pH values.^19^ Hence, to compare the features of reactive Fe(ii) surface complexes under the impact of soluble Si(iv), and further on the reduction of NACs by mineral-bound [≡Fe(iii)]/[≡Fe(ii)] couples are important for us to understand the interfacial reactions among silica, aluminum, iron and NACs in real subsurface environments.

Electrochemical methods including cyclic voltammetry (CV) and electrochemical impedance spectrometry (EIS), which can be used to determine the electron transfer rate of Fe(ii)-to-Fe(iii) as well as the magnitude of the redox potential response to the variation of the mineral-bound [≡Fe(iii)]/[≡Fe(ii)] couples, have been applied to study electron transfer reactions on the surfaces of Fe-free^5,7,8^ and Fe-contained minerals,^4,6^ and in complex systems such as real soil,^14^ and Fe(ii)/Cu(ii) interaction systems.^20–22^ The electrochemical evidences obtained from the above systems reveal that the enhanced reductive reactivity of Fe(iii)/Fe(ii) couple on mineral surfaces mainly depended on two key factors, *i.e.*, (1) the reduced peak oxidation potential (*E*_p_), and (2) the reduced charge transfer resistance (*R*_CT_) of the Fe(ii) surface complex.^8^

In addition to the electrochemical characteristics of the Fe(ii) surface complex, the reductive reactivity of the Fe(ii) surface complex is also determined by the speciation of the Fe(ii) surface complex. Previous studies revealed that the enhanced reductive reactivity of the Fe(ii) surface complex on individual Fe-free minerals, such as γ-Al_2_O_3_ and TiO_2_, is positively correlated to the concentration of the ≡Al_st_OFe^+^ and ≡TiOFe^+^ complexes,^7,8^ which were determined using surface complexation models, such as the diffuse double layer (DDL) model.^23^ However, in systems containing a Fe-contained mineral, it is difficult to determine the speciation of adsorbed Fe(ii) and predict key active adsorbed Fe(ii) species, because Fe(ii) is not only adsorbed on the Fe(iii)-containing mineral surface but also exchanges/reacts with Fe(iii) in the underlying Fe(iii)-containing mineral.^24^ This task becomes even more difficult in complex systems, such as real environmental or biogeochemical interfaces. Nevertheless, this difficulty may be overcome using Fe *K*-edge X-ray absorption spectroscopy (XAS) because it is an element-specific, sensitive and nondestructive technique, and seem to be the ideal method to solve the limitations described above.

In this study, γ-Al_2_O_3_ was selected as the sole mineral surface, and the experiments were designed and conducted to determine the amount of Fe(ii) adsorbed onto γ-Al_2_O_3_ under different Si(iv) concentrations and subsequently investigate the reduction rate of 2-nitrophenol (2-NP) at different conditions. γ-Al_2_O_3_-modified glass carbon (γ-Al_2_O_3_/GC) electrodes^8^ were prepared to investigate the electrochemical response of the [≡Fe(iii)]/[≡Fe(ii)] couples, Fe *K*-edge X-ray absorption was applied to identify the characteristics of the [≡Fe(iii)]/[≡Fe(ii)] couples on γ-Al_2_O_3_-at different Si(iv) concentrations. With the results, this study provided an insight into the influence of Si(iv) on the reductive reactivity of [≡Fe(iii)]/[≡Fe(ii)] couples for 2-nitrophenol reduction in γ-Al_2_O_3_ suspensions.

## 2. Materials and methods

### 2.1 Fe(ii) adsorption on γ-Al_2_O_3_

The experiments of Fe(ii) (FeCl_2_, >99.0%, Acors) adsorption onto γ-Al_2_O_3_ (Alu-C, 13 nm, Degussa) were conducted in borosilicate glass serum bottles (effective volume = 20 mL) using the methods described in previous studies.^7,8^ Two experimental setups were designed to investigate the influence of the adsorption order on the adsorbed Fe species: (i) Fe(ii) adsorption onto γ-Al_2_O_3_ after Si(iv) (Na_2_SiO_3_·5H_2_O, >99.0%, Acors) adsorbed onto γ-Al_2_O_3_ for 24 h (C1) and (ii) Fe(ii) and Si(iv) adsorption onto γ-Al_2_O_3_ at the same time for 24 h (C2). All experiments were performed in triplicate and were replicated three times simultaneously. The details of the experimental procedures are described in Section 1 in ESI.†

### 2.2 2-NP reduction kinetics

Under the conditions identical to those of adsorption experiments, 5.5 μM of 2-NP (>99.0%, Acors) was added to each of the reactors containing above mentioned Fe(ii)/Si(vi)–γ-Al_2_O_3_ to start the experiments of 2-NP reduction. The pH values of the kinetic measurements were controlled within a narrow range, *i.e.*, pH 6.0–7.0. Meanwhile, to further investigate the effects of the Si(iv) on the reductive transformation of 2-NP, a series of experiments was designed and conducted at pH 6.9 under different temperatures (288–318 K).

### 2.3 Analyses

The concentrations of 2-NP were determined using HPLC.^8^ The Fe(ii) and Si concentrations were determined using the 1,10-phenanthroline method at 510 nm (ref. 25 and 26) and the molybdenum blue colorimetric method,^27^ respectively. The details of the analysis methods are presented in Section 2 in ESI.†

### 2.4 Electrochemical tests

Cyclic voltammograms (CVs) were conducted in a standard three-electrode cell and recorded with an Autolab potentiostat (PGSTAT 30, Eco Chemie, The Netherlands) at a scan rate of 50 mV s^−1^.^7,8^ More detail electrochemical analysis procedures are presented in Section 3 in ESI.†

### 2.5 Fe *K*-edge X-ray absorption spectroscopic analysis

After the adsorption equilibrium, the suspensions were immediately transferred to the vacuum freeze-drying machine for at least 48 h to prepare the adsorbed powdered samples at various conditions. Each powdered sample was grinded and loaded into a Teflon sample holder. The Fe *K*-edge X-ray absorption spectra were conducted at beamline 17C1 at the National Synchrotron Radiation Research Center (NSRRC) in Hsinchu, Taiwan. For each samples, at least three scans were recorded in fluorescent mode using a Lytle detector with a 6 μm Mn filter and a set of Soller slits and the spectrum of metallic Fe was measured simultaneously with the samples for the purpose of energy calibration. The scans for each sample were then averaged, followed by background removal and normalization, using the Athena software.^28^ Additionally, the Fe *K*-edge X-ray absorption spectra were also measured for the reference samples, including Fe(ii) adsorbed onto γ-Al_2_O_3_ (S1) and Fe(ii) adsorbed onto SiO_2_ (S2), using the same sample procedure. These reference spectra were then used to conduct linear combination fitting (LCF) of the EXAFS spectra of the samples in Athena, and no energy shifts in the reference spectra were allowed in the LCF algorithm.

## 3. Results

### 3.1 pH effects and adsorption model of Fe(ii) and Si(iv) adsorbed onto γ-Al_2_O_3_

The adsorption behaviors of Fe(ii) and Si(iv) on γ-Al_2_O_3_ under different Si(iv) concentrations exhibited similar pH-dependent patterns (Fig. S1A in ESI†). The adsorptions of co-added Fe(ii) and Si(iv) both increased with increasing pH, indicating the positive effects of pH on the adsorptions of Fe(ii) and Si(vi) on the mineral. Meanwhile, the kinetic curves of Fe(ii) and Si(iv) adsorbed onto γ-Al_2_O_3_ at pH 6.9 under equal concentrations are shown in Fig. S1B in ESI.† It should be noted that the adsorption of Fe(ii) and Si(iv) onto γ-Al_2_O_3_ exhibited similar time-dependent patterns showing that the adsorption processes occurred quickly in the beginning and gradually leveled off. The adsorption equilibrium time for Fe(ii) and Si(iv) under different conditions was approximately 6 h. Furthermore, the quantity of adsorption (*Q*_*t*_) decreased both for Fe(ii) and Si(iv) compared to the adsorption of either Fe(ii) or Si(iv).

On the other hand, when equal amounts of Fe(ii) and Si(iv) coexisted in the γ-Al_2_O_3_ suspensions, the amount of Si(iv) remaining in suspension was markedly less than the amount of Fe(ii) remaining over a 24 h time period, demonstrating that γ-Al_2_O_3_ had a larger capacity for Si(iv) adsorption than Fe(ii) adsorption at pH 6.9 (Fig. S1B in ESI†). Meanwhile, Fe(ii) and Si(iv) adsorptions onto γ-Al_2_O_3_ followed the pseudo second-order model (Table S1 and Fig. S2 in ESI†).

### 3.2 Effects of Si(iv) on the reductive transformation of 2-NP by adsorbed Fe(ii)

**Fig. 1:**
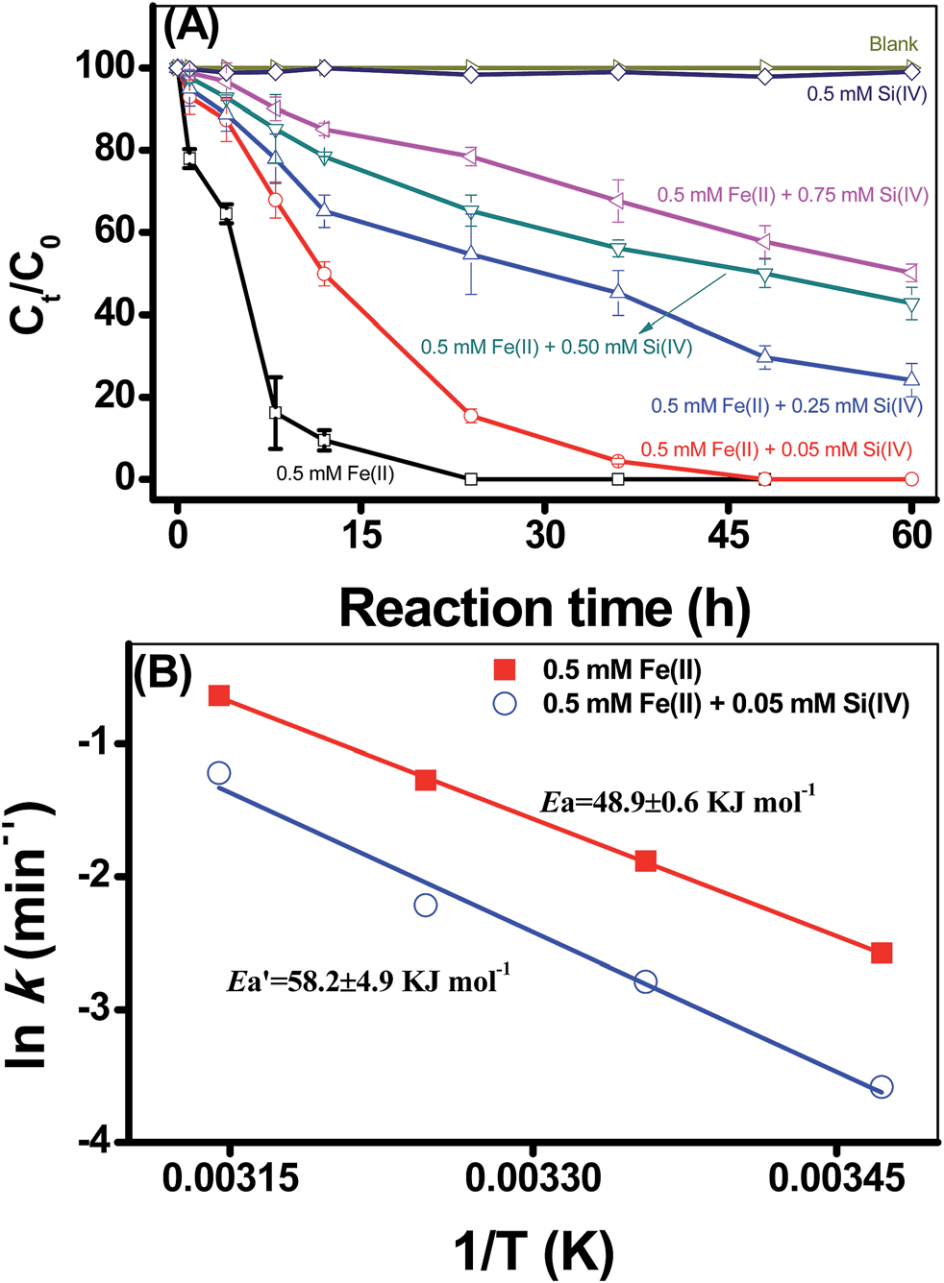
The kinetic curves of 2-NP transformation under different Si(iv) concentrations (A). The plot of ln *k* against temperature (B). Reaction conditions: 0.5 mM Fe(ii), 0 to 0.75 mM Si(iv), 5.5 μM 2-NP, 4.0 g L^−1^ mineral, pH 6.9, and 288–318 K.

Using 2-NP as the reduction probe compound, the kinetics of 2-NP reduction by adsorbed Fe(ii) on γ-Al_2_O_3_ were evaluated under different experimental conditions (Fig. 1, S3 and S4 in ESI†). 2-NP was used as the reduction probe compound because our previous studies have proved that the adsorption of 2-NP onto different minerals was very low,^4–8,14,17,21^ and thus, 2-NP adsorption has negligible contribution to the decrease of 2-NP concentration in the systems under investigation. On the other hand, the impact of pH on the reductive transformation of 2-NP demonstrated the dependencies of 2-NP degradation on the solution pH; that is, the rates of 2-NP reduction by Fe(ii) were significantly accelerated by the increase of pH (Fig. S3 in ESI†). Previous study illustrated that 2-NP reduction proceeded through intermediates to 2-aminophenol^21^ and generally followed the pseudo-first-order kinetic rate law in media consisting of Fe(ii), 2-NP and minerals. The calculated reduction rates (*k*) using pseudo-first-order rates were 0.32 × 10^−2^, 0.42 × 10^−2^, and 8.74 × 10^−2^ h^−1^ for pH values that ranged from 6.0 to 6.9; hence, the pH values of the experiments were controlled at a pH of 6.9.

Fig. 1A presents the effects of the Si(iv) concentration on the reductive transformation of 2-NP by the adsorbed Fe(ii) on γ-Al_2_O_3_. No visible 2-NP transformation was detected with the addition of Si(iv) alone, indicating that the Si(iv) species could not reduce 2-NP. Moreover, the Si(vi) concentration has a negative effect on the reduction rate of 2-NP by the adsorbed Fe(ii) on γ-Al_2_O_3_ (Fig. 1A). With the increase in the Si(iv) concentration from 0 to 0.75 mM, the calculated *k* value of 2-NP reduction decreased from 19.9 × 10^−2^ to 1.13 × 10^−2^ h^−1^ (Table 1). Thus, the existence of Si(vi) in the systems inhibited the reduction of 2-NP. Meanwhile, Fig. S4 in ESI† present the effects of temperature on the reductive transformation of 2-NP by the adsorbed Fe(ii) on γ-Al_2_O_3_ with different Si(vi) concentrations at pH 6.9. It should be noted that the reductive transformation of 2-NP sped up with an increase in the reaction temperature both with only Fe(ii) (Fig. S4A in ESI†) and with Fe(ii) and Si(iv) (Fig. S4B in ESI†), indicating that the reduction of 2-NP was an endothermic reaction.

**Table 1:** The relationships among the concentrations of the adsorption density, kinetics of 2-NP transformation, and electrochemical parameters. The pH was controlled at 6.9 in 0.2 M NaCl and 28 mM MOPS solution

Si(iv) concentration (mM)	0	0.05	0.25	0.50	0.75
*ρ*_Fe(II)_ (μM m^−2^)^a^	0.268 ± 0.015	0.247 ± 0.007	0.242 ± 0.016	0.211 ± 0.003	0.209 ± 0.018
*ρ*_Si(IV)_ (μM m^−2^)^a^	0	0.078 ± 0.001	0.432 ± 0.002	0.836 ± 0.001	1.151 ± 0.055
Rate constant of 2-NP reduction (*k* × 10^−2^ h^−1^)^b^	19.9, *R*^2^ = 0.96	8.74, *R*^2^ = 0.98	2.43, *R*^2^ = 0.99	1.50, *R*^2^ = 0.99	1.13, *R*^2^ = 0.98
Peak oxidation potential (*E*_p_/mV) (*vs.* SCE)^c^	4	9	18	23	44
Normalized *k*/Fe(ii)_sorbed_ (h^−1^ m M^−1^)^d^	1.458	0.693	0.197	0.140	0.105

a Data from Fig. 3.

b Data from Fig. 1.

c Data from Fig. 2.

d Data from Fig. 3.

The results in Fig. S4† also showed that the addition of Si(iv) decreased the reductive transformation of 2-NP under all of the reaction temperatures. The Arrhenius equation (formula (1)) can be applied to describe the temperature dependence of the reaction rate.1
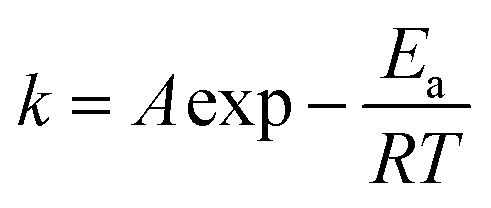
where *A* is the frequency factor, *E*_a_ is the activation energy, *R* is the universal constant and *T* is the absolute temperature. By plotting ln *k versus* 1/*T*, both the *E*_a_ and *A* can be calculated from the slope and intercept, respectively. Accordingly, the calculated results showed that the *E*_a_ value for the 2-NP reduction was 48.9 ± 0.6 kJ mol (only Fe(ii)), while the value increased to 58.2 ± 4.9 kJ mol with the addition of 0.05 mM Si(iv) (Fig. 1B). These calculations clearly indicated that the addition of Si(iv) inhibited the reductive transformation of 2-NP by increasing the *E*_a_ value of that reaction.

### 3.3 Electrochemical evidence of the Si(iv) influence on 2-NP reduction

**Fig. 2:**
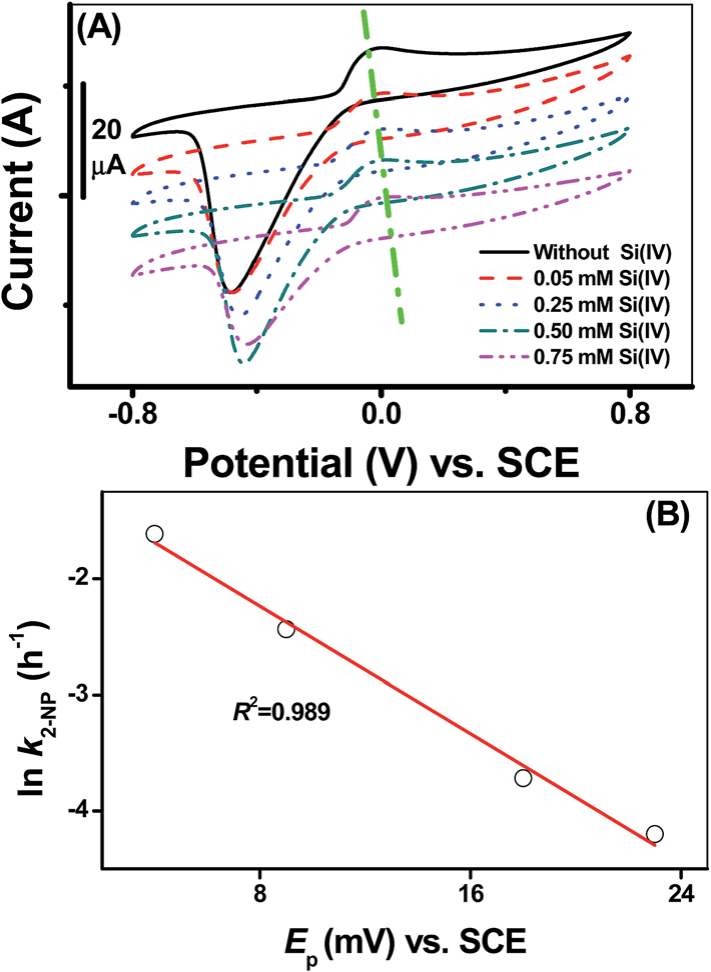
Cyclic voltammograms of adsorbed [≡Fe(iii)]/[≡Fe(ii)] couples on the γ-Al_2_O_3_/GC electrode (A). Correlations between the *E*_p_ values of the adsorbed [≡Fe(iii)]/[≡Fe(ii)] couples and the ln *k* values of 2-NP reduction (B). Electrochemical measurements were conducted in a cell (25 mL) containing 0.5 mM FeCl_2_, 0.2 M NaCl solution and 28 mM buffer at 298 K. The mineral loading was estimated to be 2 × 10^−3^ g L^−1^. The scan rate was 50 mV s^−1^.

Cyclic voltammograms of the adsorbed [≡Fe(iii)]/[≡Fe(ii)] couples onto the γ-Al_2_O_3_ modified GC electrode at various Si(iv) concentrations provided direct evidence of the change in its redox behavior, as shown in Fig. 2A. All CVs exhibited a pair of peaks: a cathodic Fe(iii) reduction peak at potentials ranging from −0.6 V to −0.4 V (*versus* SCE) and an anodic Fe(ii) oxidation peak at potentials ranging from 0 V to 0.05 V (*versus* SCE). There was a clearly visible positive shift in the peak oxidation potential (denoted as *E*_p_) of the adsorbed [≡Fe(iii)]/[≡Fe(ii)] couples as a function of the Si(iv) concentration in the systems. Based on the linear free-energy relationship (LFER), previous reports^23^ have demonstrated that the changing in Gibbs free-energy had a pronounced effect on the reduction kinetics of an organic pollutant. In general, the negative shift in the Fe(ii) oxidation potential thermodynamically reflected the shift of the Gibbs free energy to a negative value. Accordingly, the relationship between the ln *k* of the 2-NP transformation and *E*_p_ (*versus* SCE) presents a good linear correlation as indicated by the high *R*^2^ value (0.989) in Fig. 2B. Combined with the calculated *E*_a_ values obtained in Fig. 1B, it can be concluded that the positive shift in the Fe(ii) oxidation potential in the presence of Si(iv) results in an increase in the *E*_a_ value, which accounted for the inhibition of the transformation rates of 2-NP.

## 4. Discussion

### 4.1 Relationship between the 2-NP reduction rate and the adsorbed Fe(ii) density

**Fig. 3:**
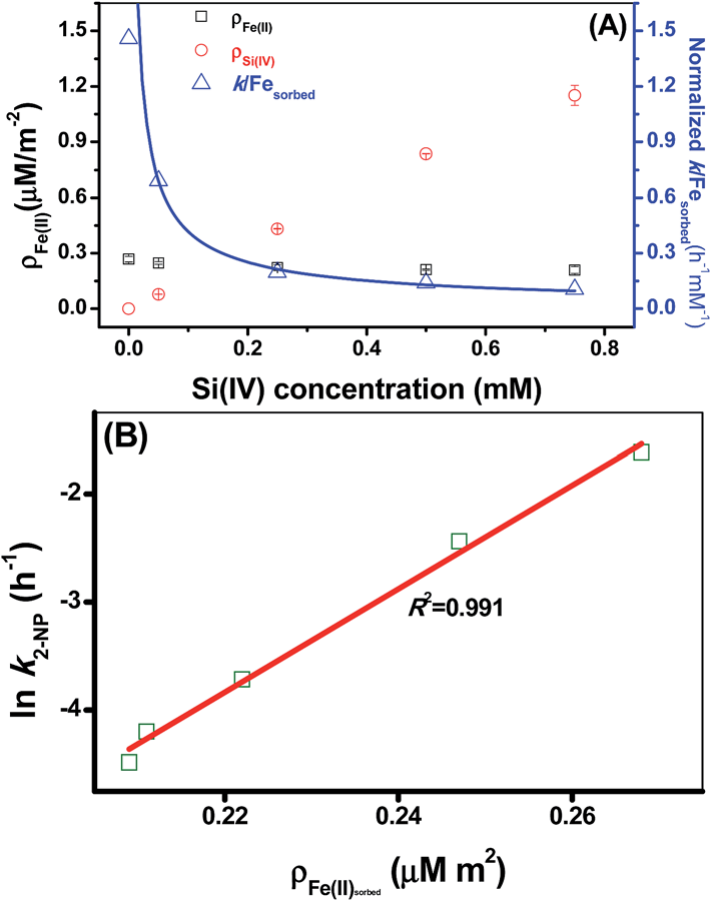
(A) Comparison between *k* normalized to the amount of adsorbed Fe(ii) for γ-Al_2_O_3_ as a function of the Si(iv) concentration. (B) Correlations between the adsorbed Fe(ii) density (*ρ*_Fe(II)_) and the ln *k* values of 2-NP reduction.

The results of the adsorption experiments at a fixed pH and Fe(ii) concentration with varying initial Si(iv) concentrations suggested that the fractional adsorption of the Fe(ii) species slightly declined whereas the fractional adsorption of the Si(iv) species linearly increased with an increase in the Si(iv) concentration (Fig. 3A). In comparison, the adsorbed Si(iv) (*ρ*_Si(IV)_) was found to be more sensitive to the Si(iv) concentration change than the adsorbed Fe(ii) (*ρ*_Fe(II)_) (Table 1 and Fig. 3A). Fig. 1A illustrates that the Si(iv) species had no reductive activity in 2-NP reduction, while the formation of the Fe(ii) surface complex enhanced the transformation rate of 2-NP.^9–12^ Hence, by normalizing the *k* values of 2-NP reduction with *ρ*_Fe(II)_, the obtained results clearly showed that the value of the normalized *k*/*ρ*_Fe(II)_ declined as the Si(iv) concentration increased (Fig. 3A). This indicated the negative effect of Si(iv) on the 2-NP reduction by Fe(ii) on γ-Al_2_O_3_. Furthermore, besides the negative linear correlation between the ln *k* of the 2-NP transformation and *E*_p_ values (Fig. 2B), a positive linear correlation (Fig. 3B) existed between the *ρ*_Fe(II)_ and ln *k* of the 2-NP transformation, as indicated by the high *R*^2^ value (0.991), illustrating that the amount of adsorbed Fe(ii) on the mineral surface was a crucial factor that affected the 2-NP reduction rate.

### 4.2 Thermodynamic modify of the adsorbed Fe(ii) redox reactivity in the presence of Si(iv)

The observed *E*_p_ values were the electrode potentials of the adsorbed [≡Fe(iii)]/[≡Fe(ii)] couples, which could be described by the Nernst equation at 298 K (formula (2)).2
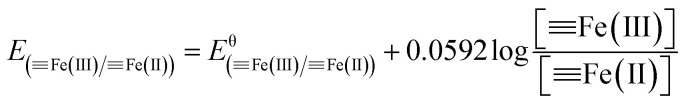
where *E*^θ^_(≡Fe(III))/(≡Fe(II))_ is the standard electrode potential of the [≡Fe(iii)]/[≡Fe(ii)] couples, and the *E* values of the adsorbed [≡Fe(iii)]/[≡Fe(ii)] couples in heterogeneous suspensions are usually decided by the ratio of the adsorbed [≡Fe(iii)]/[≡Fe(ii)] couples. The characteristics of the adsorbed [≡Fe(iii)]/[≡Fe(ii)] couples in the reductive transformation of 2-NP have been studied on various interfaces (*i.e.*, Fe-free minerals and Fe-contained minerals) and the observed *E*_p_ values of the different adsorbed [≡Fe(iii)]/[≡Fe(ii)] couples on those various minerals presented significant differences, even under the same experimental conditions.^4,8,17^ In general, the overall trend for *ρ*_Fe(II)_ at all of the reaction pH values was larger for the Fe-contained mineral surfaces than those values on the Fe-free mineral surfaces;^4^ the Fe-contained mineral surface-bound Fe(iii)/Fe(ii) couples generally possessed a more negative *E*_p_ compared to the Fe-free mineral surface-bound Fe(iii)/Fe(ii) couple, leading to a higher reaction rate.^4^ Additionally, the adsorbed [≡Fe(iii)]/[≡Fe(ii)] couples on γ-Al_2_O_3_ had a lower *E*_p_ value than that on SiO_2_ and had higher *k* values for the reduction of 2-NP on γ-Al_2_O_3_ than that on SiO_2_.^8,29^ Accordingly, we hypothesized that modification of the adsorbed [≡Fe(iii)]/[≡Fe(ii)] couples with an increase in Si(iv) would lead to a positive shift in *E*_p_ and decrease in *ρ*_Fe(II)_, which consequently decreased the *k* value for the reduction of 2-NP.

### 4.3 The modification of the adsorbed [≡Fe(iii)]/[≡Fe(ii)] couples proved by Fe *K*-edge XAS spectroscopy

**Fig. 4:**
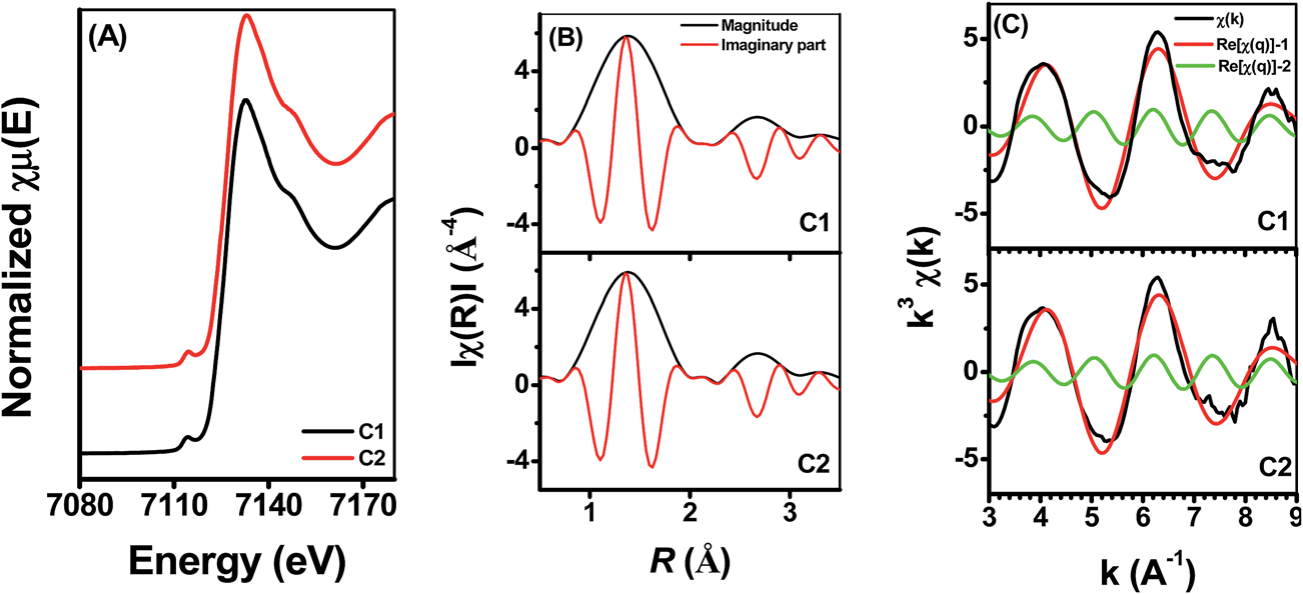
Fe *K*-edge XANES spectra (A), *R* space (B), and the Fe *K*-edge *k*^3^-weighted EXAFS spectra (C) under different adsorption orders at pH 6.9.

Fe *K*-edge X-ray absorption spectra were applied to investigate the characteristics of the adsorbed [≡Fe(iii)]/[≡Fe(ii)] couples when the Si(iv) concentration was increased. Fig. 4 presents the obtained results of (i) Fe(ii) adsorption onto γ-Al_2_O_3_ after Si(iv) sorbed onto γ-Al_2_O_3_ for 24 h (C1) and (ii) Fe(ii) and Si(iv) adsorption onto γ-Al_2_O_3_ at the same time for 24 h (C2) at pH 6.9. Under these two experimental conditions, all of the samples showed similar Fe *K*-edge XANES spectra (Fig. 4A) characteristic features: the position and shape of the pre-edge peak (7114.44 eV), the inflection point of the absorption edge (7127.34 ± 0.24 eV) and the white line (7133.04 ± 0.25 eV). Meanwhile, the *R* space (Fig. 4B) of C1 and C2 showed no significant difference between the experimental line (black) and the calculated imaginary part line (red). Furthermore, Fig. 4C shows the Fe *K*-edge *k*^3^-weighted EXAFS spectra of C1 and C2 with the contribution of the first shell (*R* = 0.8–2.2) presented by the red line and the contribution of the second shell (*R* = 2.2–3.2) presented by the green line. The fits, demonstrated by the dotted line in Fig. 4C, were in excellent agreement with the corresponding measured spectra (black line). All of the results in Fig. 4 show similar Fe *K*-edge XANES spectra, *R* space, and *k*^3^-weighted EXAFS spectra, indicating that there was no observable difference between the two experimental setups (C1 and C2). Furthermore, Fig. S5 in ESI† show the Fe *K*-edge X-ray absorption spectra of Fe adsorbed onto γ-Al_2_O_3_ with different Si(iv) concentrations at pH 6.9. With the increase of Si(iv) concentrations, the obtained results of Fe *K*-edge XANES spectra showed similar characteristic features: the position and shape of the pre-edge peak, the inflection point of the absorption edge and the white line (Fig. S4A†). Thus, the adsorption order and amount of Si(iv) had negligible influence on the bonding configuration of the adsorbed Fe species on γ-Al_2_O_3_.

**Fig. 5:**
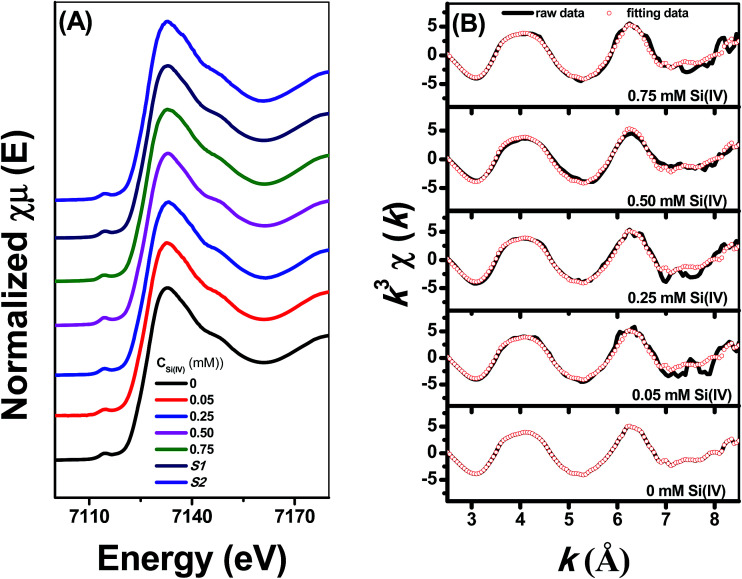
Fe *K*-edge XANES spectra (A) and the Fe *K*-edge *k*^3^-weighted EXAFS spectra (B) under different Si(iv) concentrations at pH 6.9.

Fig. 5 shows the Fe *K*-edge X-ray absorption spectra of Fe adsorbed onto γ-Al_2_O_3_ with different Si(iv) concentrations at pH 6.9. To quantitatively determine the spatial distribution of Fe under the influence of different Si(iv) concentrations, linear combination fitting (LCF) was performed for those spectra using the spectra of the reference compounds, *i.e.*, S1 (Fe(ii) adsorbed onto γ-Al_2_O_3_) and S2 (Fe(ii) adsorbed onto SiO_2_). S1 (Fe(ii) adsorbed onto γ-Al_2_O_3_) was used to account for the contribution of various bonding structures of Al–O–Fe to the measured spectra, while S2 (Fe(ii) adsorbed onto SiO_2_) was used to account for the contribution of various bonding structures of Si–O–Fe to the measured spectra. The Fe *K*-edge *k*^3^-weighted EXAFS spectra combined with the best fits of the samples are presented in Fig. 5B and summarized in Table 2. The fits, demonstrated by the dotted line in Fig. 5B, were in excellent agreement with the corresponding measured spectra. Meanwhile, the LCF results indicated a decline proportion in S1 and a rise proportion in S2 with an increase in the Si(iv) concentration. Hence, the increase trend of S2 as well as the decrease trend of S1 with the increase of the exogenous addition of Si(iv) in the reaction system illustrate the modification of the component of the adsorbed [≡Fe(iii)]/[≡Fe(ii)] couples, which lead to a decrease in *ρ*_Fe(II)_ (Fig. 3), positive shift in *E*_p_ (Fig. 2), and decrease in the *k* value observed in the reduction experiments of 2-NP.

**Table 2:** LCF results of the Fe *K*-edge EXAFS spectra under different Si(iv) concentrations

Samples	Proportion of the standards in the sample (%)	
	S1 (≡AlOFe^+^)^a^	S2 (≡SiOFe^+^)^b^
0 mM Si(iv)	1.000 (0.000)	0.000 (0.000)
0.05 mM Si(iv)	0.964 (0.031)	0.036 (0.031)
0.25 mM Si(iv)	0.808 (0.056)	0.192 (0.056)
0.50 mM Si(iv)	0.746 (0.064)	0.254 (0.064)
0.75 mM Si(iv)	0.692 (0.057)	0.308 (0.057)

a Fe(ii) sorbed onto γ-Al_2_O_3_ with 0 mM Si(iv).

b Fe(ii) sorbed onto SiO_2_ with 0 mM Si(iv).

### 4.4 Comparison of the Fe(ii) surface complex and their reductive reactivity in 2-NP transformation

**Fig. 6:**
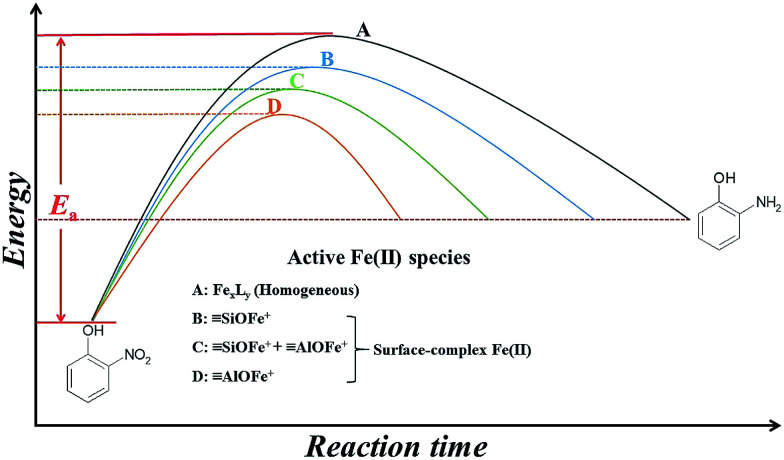
The comparison of *E*_a_ and possible active [≡Fe(iii)]/[≡Fe(ii)] couples in 2-NP transformation under different reaction conditions.

Fig. 6 presents a comparison of the activation energies as well as possible active Fe(ii) species in the 2-NP transformation under different conditions. The homogeneous suspensions (route A), of which the active Fe(ii) species were Fe_*x*_L_*y*_ (L: organic/inorganic ligands), carried the highest *E*_a_ value and the smallest *k* value.^8,9,13^ On the contrary, Fe(ii) can be stabilized by the hydroxyl groups providing by the mineral surface to form various kinds of Fe(ii) surface complexes, such as ≡SOFe^+^ and ≡SOFeOH^0^, which can lower the redox potential compared to aqueous Fe(ii) species.^9–12^ As presented in Fig. 6, the transformation of 2-NP in heterogeneous suspensions (route B, C, and D) were more quickly than those in homogenous suspensions. The enhanced 2-NP reduction rates in the heterogeneous suspensions were attributed to the adsorbed [≡Fe(iii)]/[≡Fe(ii)] couples (≡SOFe^+^). In detail, the active [≡Fe(iii)]/[≡Fe(ii)] couples on solo SiO_2_ (route B) and γ-Al_2_O_3_ (route D) were ≡SiOFe^+^ (SiO_2_ surface-bound Fe species) and ≡AlOFe^+^ (γ-Al_2_O_3_ surface-bound Fe species), respectively. The *E*_a_ value in the SiO_2_ suspensions was higher than that in the γ-Al_2_O_3_ suspensions, whereas the *k* value in the SiO_2_ suspension was lower than that in the γ-Al_2_O_3_ suspensions.^7,8,29^ In the presence of Si(iv) in γ-Al_2_O_3_ suspensions (route C), the active Fe(ii) species changed from pure ≡AlOFe^+^ to a mixture with ≡AlOFe^+^ and ≡SiOFe^+^. The presence of ≡SiOFe^+^ increased the *E*_a_ value, leading to a decrease in *ρ*_Fe(II)_, a positive shift in *E*_p_, and a decrease in the *k* value for the reduction of 2-NP. Combining the results discussed above and the results from the Fe *K*-edge X-ray absorption spectra (Fig. 5 and Table 2), the influence of Si(iv) on the reductive reactivity of the adsorbed [≡Fe(iii)]/[≡Fe(ii)] couples mainly presented by modifying the components of the active adsorbed [≡Fe(iii)]/[≡Fe(ii)] couples.

## 5. Conclusion

The reduction of 2-NP by adsorbed Fe complex mainly depended on two key issues: (1) the value of *ρ*_Fe(II)_ and (2) the value of *E*_p_ (lower *E*_p_ can enhance the 2-NP reduction reaction from the view of thermodynamics). The results of this study provided an understanding of the negative effects of Si(iv) on the reductive reactivity of the adsorbed [≡Fe(iii)]/[≡Fe(ii)] couples on a mineral surface, mainly by modifying the properties of the adsorbed [≡Fe(iii)]/[≡Fe(ii)] couples through changing the above two key factors of *ρ*_Fe(II)_ and *E*_p_ under various reaction conditions. The Fe *K*-edge XAS spectroscopy method could be a useful tool to quantitatively determine the subtle changing properties of the Fe surface complex involving in the reduction of contaminants in real subsurface environments.

## Conflicts of interest

There are no conflicts to declare.

## Supplementary Material

RA-008-C7RA13201H-s001
