# Supplementary material for: The influence of Si(iv) on the reactivity of [[triple bond, length as m-dash]Fe(iii)]/[[triple bond, length as m-dash]Fe(ii)] couples for 2-nitrophenol reduction in γ-Al2O3 suspensions
Source: RSC Adv. 2018 Feb 15;8(14):7465–72. doi: 10.1039/c7ra13201h (PMC9078414; doi:10.1039/c7ra13201h)
Supplement: RA-008-C7RA13201H-s001 [file RA-008-C7RA13201H-s001.pdf]

Electronic Supplementary Material (ESI) for RSC Advances.
This journal is © The Royal Society of Chemistry 2018

The influence of Si(IV) on the reactivity of [≡Fe(III)]/[≡Fe(II)] couples for 2-nitrophenol reduction in γ-Al_2O_3 suspensions

Liang Tao [a], Shan-Li Wang [b], Fang-Bai Li [a*], Nin-Ya Yu [c], Ke Wu [a, c]

[a] Guangdong Key Laboratory of Integrated Agro-environmental Pollution Control and Management, Guangdong Institute of Eco-environmental Science & Technology, Guangzhou 510650, P. R. China

[b] Department of Agricultural Chemistry, National Taiwan University, Taipei 10617, Taiwan，Republic of China

[c] College of Chemistry and Chemical Engineering, Hunan Normal University, Changsha 410081, P. R. China

11 Pages

8 Sections

5 Figures

1 Table

Submitted to RSC Advances

Corresponding author. Tel: 86-20-37021396; Fax: 86-20-87024123. E-mail: cefbli@soil.gd.cn (Fangbai Li)

Section S1 Adsorption Study

Batch studies were conducted to assess the influence of pH (from 4.0 to 8.0) on the extent of Fe(II) adsorption using the similar method with modification (Li et al., 2009). In detail, to prevent any Fe(II) oxidation, all experiments were conducted inside an oxygen-free glovebox (Model Bactron II, Anaerobic Chamber, 200 plate capacity, USA) at 298 K. Stock solutions of 0.5 M $FeCl_2$ (> 99.0%, Acors), 0.06 M MES (2-[N-morpholino] ethanesulfonic acid monohydrate, > 99.5%, Sigma-Aldrich), 0.06 M MOPS (3-[N-morpholino] propanesulfonic acid, > 99.5%, Sigma-Aldrich), and 0.44 M NaCl were prepared with reagent-grade water (18 MΩ cm resistivity, Milli-Q water). Equal volume of buffer (MES or MOPS) and NaCl were mixed together and the solution pH (4.0 to 8.0) was then adjusted by adding diluted HCl or NaOH solution. After that, different amount of Si(IV) was dissolved into the above solution and solutions were filtered through 0.2 μm filter prior to use. Suspensions prepared for the reactor contained 0.5 mM Fe(II), 0~0.75 mM Si(IV) ($Na_2SiO_3 \cdot 5H_2O$, > 99.0%, Acors), 28 mM buffer, 200 mM NaCl and 68.0 mg of γ-Al_2O_3 mineral powder (Alu-C, 13 nm, Degussa) were then placed on a rotator at 200 rpm and 298 K. After equilibrium, the final pH of each suspension was recorded before filtering (0.2-μm membrane filter). The acidified filtrate was then collected for analysis of the Fe(II) content.

Section S2 Analyses.

The concentration of 2-NP as a function of reaction time was monitored by HPLC 2487 (5 μm Symmetry-C18, 4.6 mm, 250 mm, Waters, USA), which consists of a Waters 1525 Binary pump, an analytical reversed-phase column and a Waters dual λ Absorbance UV/Vis detector as described elsewhere (Li et al., 2009; Tao et al., 2010). The isocratic mobile phase contained 80/20 (v/v) methanol/water and 3 mM HCl at a flow rate of 1.0 ml min^{-1} under isocratic conditions at 30 ± 1 ^{o}C and the wavelength was set at 213 nm. Concentrations of 2-NP were calculated by comparison with standard solutions [1]. For 2-NP kinetic studies, one

of the 20-ml serum bottles was taken from the shaker and transformed to the oxygen-free glovebox prior to routine analysis. After being mixed vigorously, the serum bottle was opened and spiked with 2 M HCl (< 60 μL) to adjust pH to below 3 for preventing further degradation of 2-NP. The suspension was immediately centrifuged at 10000 rpm for 10 min (Sigma-3K 15) to remove the particles and the supernates were remained for further analysis.

Section S3 Electrochemical tests.

Electrochemical tests were applied using the same methods as previous studies (Li et al., 2009; Tao et al., 2009; 2010). In detail, mineral-modified glassy carbon (GC) electrodes were prepared using bare GC electrodes with a diameter of 3 mm, in which a GC electrode was initially polished with emery paper, followed by Al_2O_3 powders (particle sizes of 0.06 and 1 μm). Between the two polishing steps, the electrode was thoroughly rinsed with deionized water. The polished electrode was sequentially cleaned with acetone and water in an ultrasonic bath for 10 min. Then, the mineral slurry containing 5 mg of γ-Al_2O_3 was prepared in a dilute Nafion solution (0.5 wt%, 250 μL) and placed in an ultrasonic bath for 15 min. By using a micro-syringe, aliquots (2 μL) of the above slurry were coated on the clean GC electrode, and the electrode with the coating was air dried for 30 min prior to use. The mineral-modified glassy carbon electrode is named "γ-Al_2O_3/GC".

Electrochemical measurements were carried out in a standard three-electrode cell, consisting of a working electrode (γ-Al_2O_3/GC), a counter electrode (platimum spiral) and a saturated calomel electrode (SCE) as the reference electrode (Li et al., 2009; Tao et al., 2009; 2010). Cyclic voltammgrams were recorded with an Autolab potentiostat (PGSTAT 30, Eco Chemie, The Netherlands) at a scan rate of 50 mV s^{-1}. The electrochemical cell was filled with a solution containing 3.0 mM $FeCl_2$ and 0.20 M NaCl buffered with 28 mM MES (2-Morpholin-4-yl-ethanesulfonic acid, hydrate) or MOPS (3-N-morpholino propansulfonic

acid) and the solution pH was adjusted by adding diluted HCl or NaOH solution. High purity nitrogen gas was bubbled through the above electrolyte to remove dissolved oxygen. CV tests were performed under nitrogen atmosphere at 25 °C.

Table S1 The kinetic fitting results of Fe(II) and Si(IV) adsorbed onto γ-Al_2O_3 at pH 6.9.

$Q_{e, exp}$ (mmol·g^{-1})			Pseudo-first-order			Pseudo-second-order		
			K_1 (min^{-1})	$Q_{e, cal}$ (mmol·g^{-1})	R^2	K_2 (g·mmol^{-1}·min^{-1})	$Q_{e,cal}$ (mmol·g^{-1})	R^2
Fe(II) adsorption	Only Fe(II)	0.049	0.274	0.038	0.992	14.085	0.053	0.999
	Fe(II)+ Si(IV)	0.043	0.245	0.025	0.949	18.603	0.046	0.998
Si(IV) adsorption	Only Fe(II)	0.099	0.176	0.033	0.848	10.478	0.101	0.988
	Fe(II)+ Si(IV)	0.090	0.207	0.022	0.730	13.923	0.092	0.988

Section S4 The quantity of adsorption (Q_t) data for Fe(II) and Si(IV) under different conditions were fit with pseudo first-order (formula 1) and pseudo second-order methods (formula 2), and the results are shown in Table S1 and Figure S2.

$$\ln\left(Q_e - Q_t\right) = \ln Q_e - k_1 t \qquad \text{(formula 1)}$$

$$t/Q_t = 1/k_2Q_e^2 + t/Q_e \qquad \text{(formula 2)}$$

where t is the adsorption time, Q_e is the equilibrium adsorption quantity, Q_t is the adsorption quantity at adsorption time t, and k_1 and k_2 are the pseudo first-order kinetic adsorption constant and the pseudo second-order kinetic adsorption constant, respectively.

As revealed by the coefficient of determination (i.e., R^2 value), the pseudo second-order model provided fit the experimental data better than the pseudo first-order model. The calculated Q_e ($Q_{e,\ cal}$) values from the pseudo second-order model were closer to the experimental Q_e ($Q_{e,\ exp}$) values than their counterparts from the pseudo first-order model under all the experimental conditions. Hence, Fe(II) and Si(IV) adsorptions onto γ-Al_2O_3 followed the pseudo second-order model.

FIGURE S1 Fractional adsorption of Fe(II) and Si(IV) on γ-Al_2O_3 as a function of pH (A). The kinetic curves of Fe(II) and Si(IV) adsorbed onto γ-Al_2O_3 under different conditions (B). Reaction conditions: 0.5 mM Fe(II), 0 to 0.75 mM Si(IV), pH 4-8, 4.0 g·L^{-1} mineral, and 298 K.

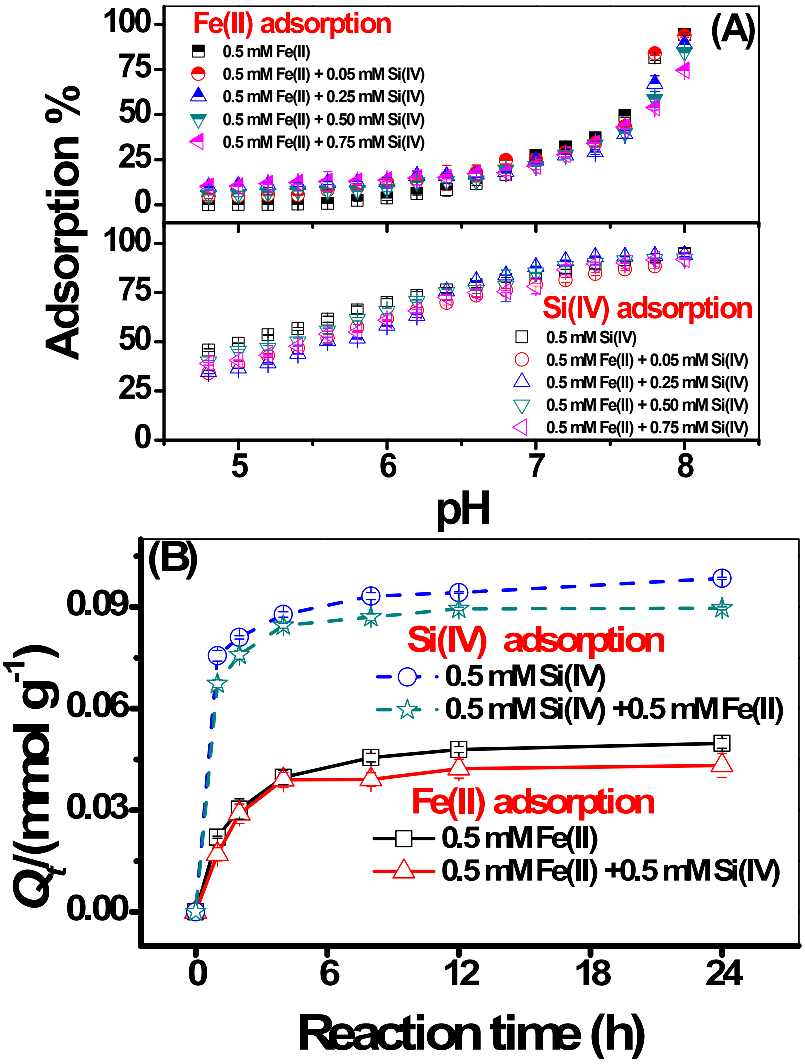

FIGURE S2 Kinetic plots fitted by pseudo first-order (A) and pseudo second-order methods (B) under different conditions. Reaction conditions: 0.5 mM Fe(II), 0.5 mM Si(IV), pH 6.9, 4.0 g·L^{-1} mineral, and 25 ^{o}C.

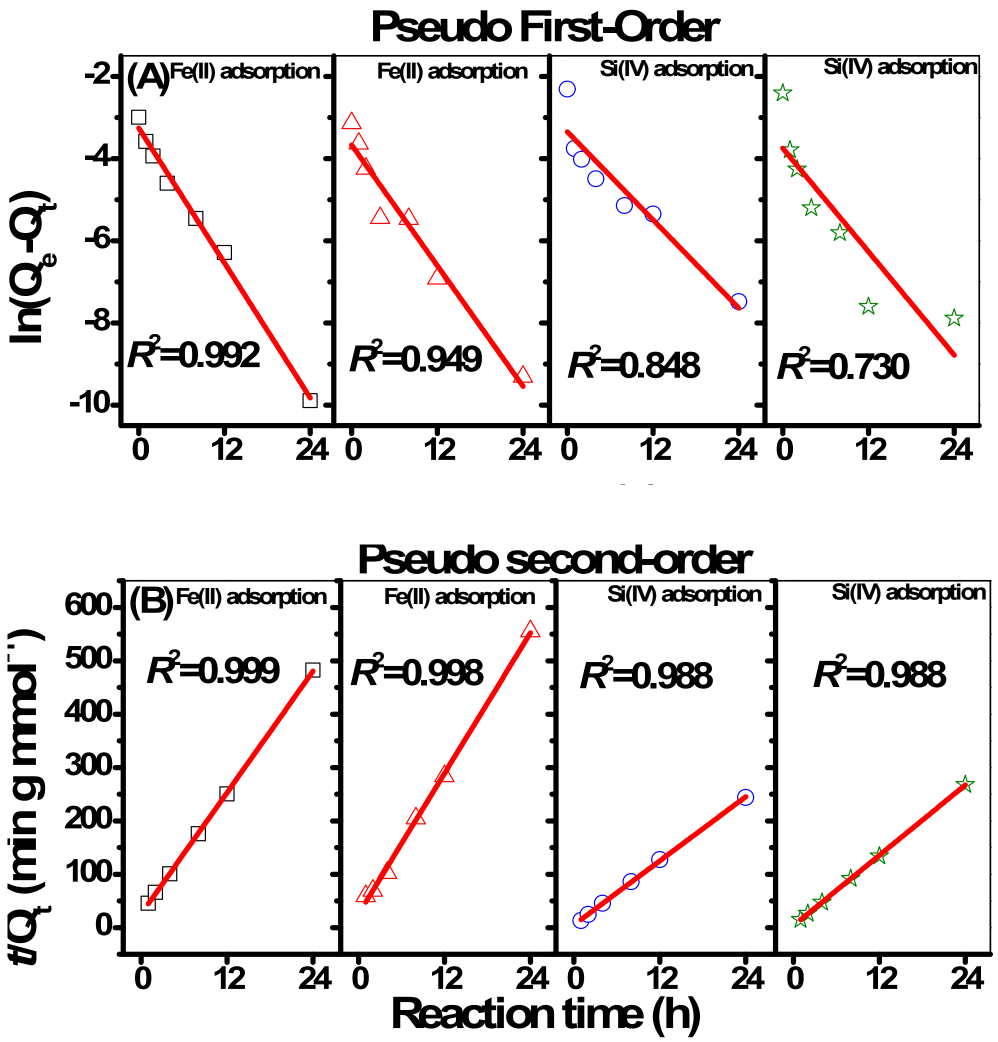

FIGURE S3. The kinetic curves of 2-NP transformation under different pH concentrations. Reaction conditions: 0.5 mmol·L^{-1} Fe(II), 5.5 μM 2-nitrophenol, 4.0 g L^{-1} mineral and 25 °C.

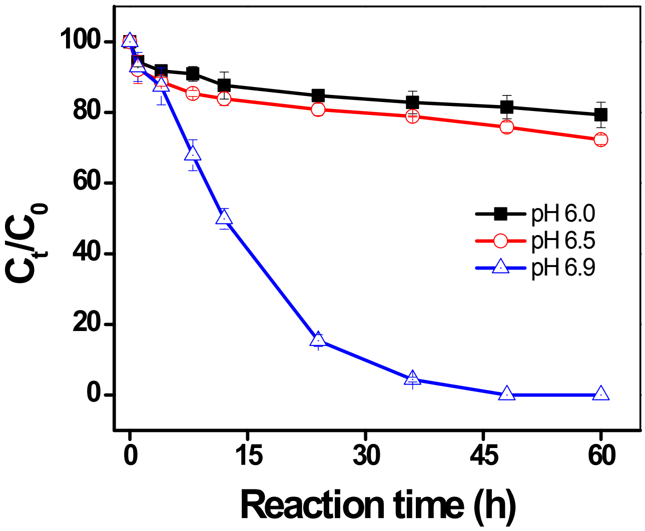

Section S5 Reduction of 2-NP in reaction media consisting of Fe(II) and minerals were studied at pH 6.7 and 25 °C. Figure S2 shows the comparison of reaction kinetics obtained under different pH conditions. Apparently, the rates of 2-NP reduction by Fe(II) were significantly enhanced with an increase in pH. Further analysis of the linear relationship between $\ln\frac{[2-NP]_t}{[2-NP]_0}$ and reaction time reveals that the transformation of 2-NP generally follows the pseudo-first-order kinetic rate law in the media consisting of Fe(II), 2-NP and minerals. That is,

$$\ln\frac{[2-NP]_t}{[2-NP]_0} = -kt \quad (1)$$

where $[2\text{-NP}]_0$ and $[2\text{-NP}]_t$ (M) are concentrations of 2-NP detected at the initial time and the reaction time, respectively; k (min^{-1}) value is the pseudo-first-order kinetic constant for 2-NP transformation; and t (min) is the reaction time. The calculated reduction rate (k) using pseudo -first-order rate was 0.32×10^{-2}, 0.42×10^{-2}, and $8.74\times10^{-2}/h^{-1}$, respectively.

FIGURE S4 Effects of temperature on the transformation of the 2-NP in γ-Al_2O_3 suspension under different conditions with Fe(II) (A), and Fe(II) with Si(IV) (B). Reaction conditions: 0.5 mmol·L^{-1}Fe(II), 0 to 0.75mmol·L^{-1} Si(IV), 5.5 μmol·L^{-1} 2-NP, 4.0 g L^{-1} mineral, pH 6.9, and 288-318 K.

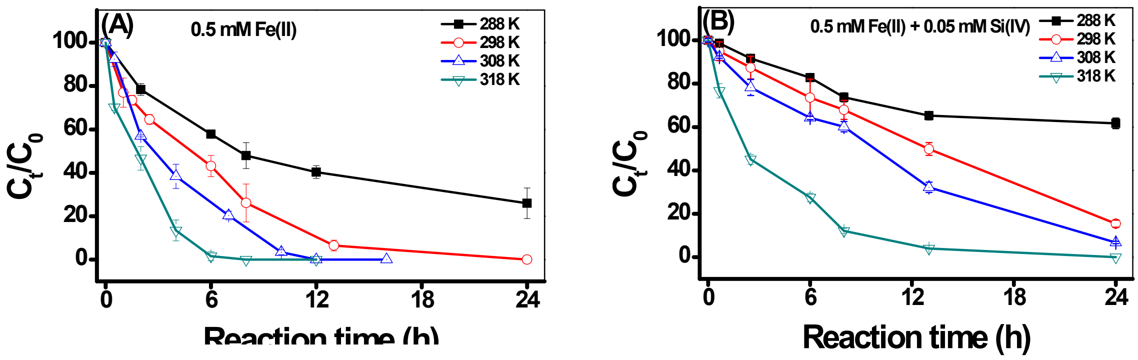

Section S6 Figure S4 shows the effects of Si(IV) concentration on the reductive transformation of 2-NP under different temperatures. The calculated *k* values was increased from 7.61× 10^{-2} to 52.7× 10^{-2} h^{-1} (with only Fe(II)) and from 2.78× 10^{-2} to 29.6× 10^{-2} h^{-1} (with Fe(II) and Si(IV)) with the increase of temperature from 288 to 318K, respectively, indicating the reduction of 2-NP is a endothermic reaction. The obtained results also indicate that the addition of Si(IV) decreased the reductive transformation of 2-NP under all the reaction temperatures.

FIGURE S5. The Fe *K*-edge XANES spectra (A), R space (B), and the Fe *K*-edge k^3-weighted EXAFS spectra (C) under different Si(IV) concentrations at pH 6.9.

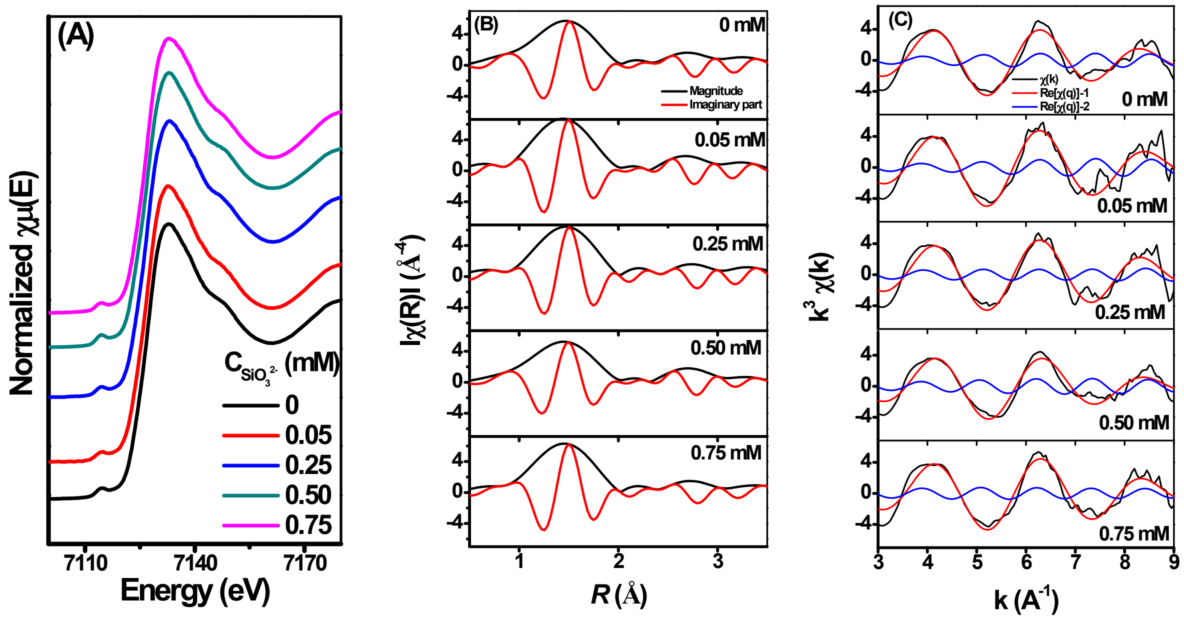

Section S7 Under these different Si(IV) concentrations, all of the samples showed similar Fe *K*-edge XANES spectra (Figure S4A) characteristic features: the position and shape of the pre-edge peak, the inflection point of the absorption edge and the white line. Meanwhile, the *R* space (Figure S4B) of these samples showed no significant difference between the experimental line (black) and the calculated imaginary part line (red). Furthermore, Figure S4C shows the Fe *K*-edge k^3-weighted EXAFS spectra of these samples with the contribution of the first shell (R=0.8~2.2) presented by the red line and the contribution of the second shell (R=2.2~3.2) presented by the green line. The fits, demonstrated by the dotted line in Figure S4C, were in excellent agreement with the corresponding measured spectra (black line).

Section S8 REFERENCES

Li, F., Tao, L., Feng, C., Li, X., Sun, K., 2009. Electrochemical Evidences for Promoted Interfacial Reactions: The Role of Fe(II) Adsorbed onto γ-Al2O3and TiO2in Reductive Transformation of 2-Nitrophenol. Environmental science & technology 43, 3656-3661.

Tao, L., Li, F., Feng, C., Sun, K., 2009. Reductive transformation of 2-nitrophenol by Fe(II) species in γ-aluminum oxide suspension. Applied Clay Science 46, 95-101.

Tao, L., Li, F.B., 2010. Interaction between reductive transformation of 2-nitrophenol and adsorbed Fe(II) species. Proceedings of the 19th World Congress of Soil Science: Soil solutions for a changing world, Brisbane, Australia, 1-6 August 2010., 86-89.
